# Pseudo-aortic dissection after sudden cardiac death in coronary angiography a case report: Pearls and pitfalls in false aortic dissection artifacts

**DOI:** 10.1016/j.ijscr.2022.107659

**Published:** 2022-09-14

**Authors:** Artemio García-Escobar, Silvio Vera-Vera, Alfonso Jurado-Román, Santiago Jiménez-Valero, Guillermo Galeote, Raúl Moreno

**Affiliations:** Cardiology Department, Interventional Cardiology Division, La Paz University Hospital, Madrid, Spain; Hospital La Paz Institute for Health Research (IDIPAZ), Spain; Biomedical Research Network Center on Cardiovascular Disease, Institute of Health Carlos III, Spain

**Keywords:** Pseudo-aortic dissection, False-positive aortic dissection, Cardiac arrest, Coronary angiography

## Abstract

**Introduction and importance:**

Various artifacts mimicked aortic dissection, such as streak artifacts generated by high-attenuation material, high-contrast interfaces, cardiac motion, periaortic structures, aortic wall motion, and normal aortic sinuses, have been described in the literature. Most artifacts that simulate ascending aortic dissection occur frequently on conventional CT. Their position is predictable and is related to systolic aortic motion. However, so far, to the best of our knowledge, this is the first pseudo-aortic dissection reported during coronary angiography in cardiac arrest.

**Case presentation:**

We report a case of a middle-aged man transferred to our hospital after an out-of-hospital cardiac arrest. The coronary angiography revealed non-obstructive coronary arteries and an image of probable aortic dissection was observed. Given the persistent asystole despite a prolonged advance cardiopulmonary resuscitation and the possibility of aortic dissection, a prompt in-room heart team discussion was performed. It was decided to stop and withdraw potentially life-sustaining treatment due to futility. The necropsy study revealed the aorta with some mild atherosclerotic plaques but without either aneurysm or thrombosis. The coronary arteries were reported as with patency, but in the proximal left anterior descending artery (LAD), the intima layer presented a thickness that decreased 50 % of the luminal area without signs of complicated acute plaques.

**Clinical discussion:**

In this case, the systolic aortic motion theory cannot explain the false-aortic dissection image in the coronary angiography because the patient was under cardiac arrest. Studies with arterial and venous pressures devices recording in cardiac arrest, demonstrated an abnormal hemodynamic flow, suggesting that the hemodynamic flow might be backward during cardiopulmonary resuscitation Therefore, in the setting of this abnormal hemodynamic flow, the injection of contrast may have an abnormal distribution and flow in the aorta creating an image of pseudo-aortic dissection.

**Conclusion:**

Although the exact mechanism of this false-positive aortic dissection in cardiac arrest remains unknown, operators should be aware of this entity during coronary angiography in the setting of cardiac arrest with mechanical chest compressions to avoid diagnostic errors in clinical practice.

## Highlights

• Most artifacts that simulate ascending aortic dissection are related to systolic aortic motion.
• In cardiac arrest with mechanical cardiac compressions, venous pressure is dominant to arterial.
• Hemodynamic flow might be backward during cardiopulmonary resuscitation.
• False-positive aortic dissection can be seen in cardiac arrest with mechanical cardiac compressions.

## 1 Introduction

A variety of other artifacts mimicked aortic dissection, such as streak artifacts generated by high-attenuation material, high-contrast interfaces, cardiac motion, periaortic structures, aortic wall motion, and normal aortic sinuses, have been described in the literature [1]. However, so far, to the best of our knowledge, this is the first pseudo-aortic dissection reported during coronary angiography in cardiac arrest. Through the literature review, we will highlight the mechanisms of false-positive aortic dissection and the abnormal hemodynamic flow in cardiac arrest. In addition, we will provide the pearls and pitfalls to identify this entity in order to prevent diagnostic errors in clinical practice.

## 2 Case presentation

A 51-year-old male was transferred to our hospital after an out-of-hospital cardiac arrest. He had family history of sudden cardiac death in one brother with autopsy study that reported normal cardiac findings, and genetic test was not performed. Moreover, he had a body mass index of 28.5, and medical history of uncomplicated renal colic, otherwise there was no more relevant medical history in their electronic health record system. In this regard, his family denied medical history of hypertension, diabetes, dyslipidemia, allergies, smoking and illicit drugs. In addition, his family states that he used to drink 1–2 alcoholic drinks each week. He had no prescription medication, over-the-counter medications or supplements. Regarding his social history, he was of Caucasian and Hispanic ethnicity, and his occupation was computer engineering. Based on his income, he was on middle social class. He lived independently with his wife and two children. Furthermore, he had received the first dose of the vaccine Ad26.COV2.S (Janssen®) against COVID-19 48 h ago. His family admitted that the patient stated asthenia, myalgias, atypical chest discomfort, and low-grade fever (37,4 °C) in the last 24 h.

The patient had a witnessed collapse by his son, and basic cardiopulmonary resuscitation was started by the local police. An automated external defibrillator advised shock, and he was defibrillated twice. Then, 15 min later, paramedics arrived and found the patient in ventricular fibrillation, advanced cardiopulmonary resuscitation was performed by mechanical chest compressions (Lucas®), pulse activity was restored, an ECG showed atrial fibrillation with ST elevation in V1-V2, AVR, and diffuse ST depression in all the leads. During ambulance transportation, the patient had several ventricular fibrillation episodes that were defibrillated and finally came out in asystole. After approximately 1.5 h since the patient experienced a sudden cardiac arrest and 75 min of advanced cardiopulmonary resuscitation, of which 45 min were in asystole, the patient arrived at our center. On admission, the patient was unconscious and in asystole rhythm. Bedside echocardiography ruled out pericardial effusion and pneumothorax.

**Fig. 1:**
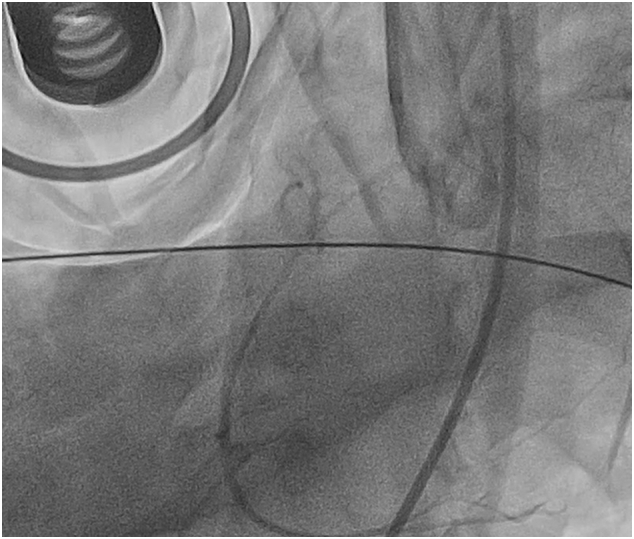
An image of probable aortic dissection in the coronary angiography.

**Fig. 2:**
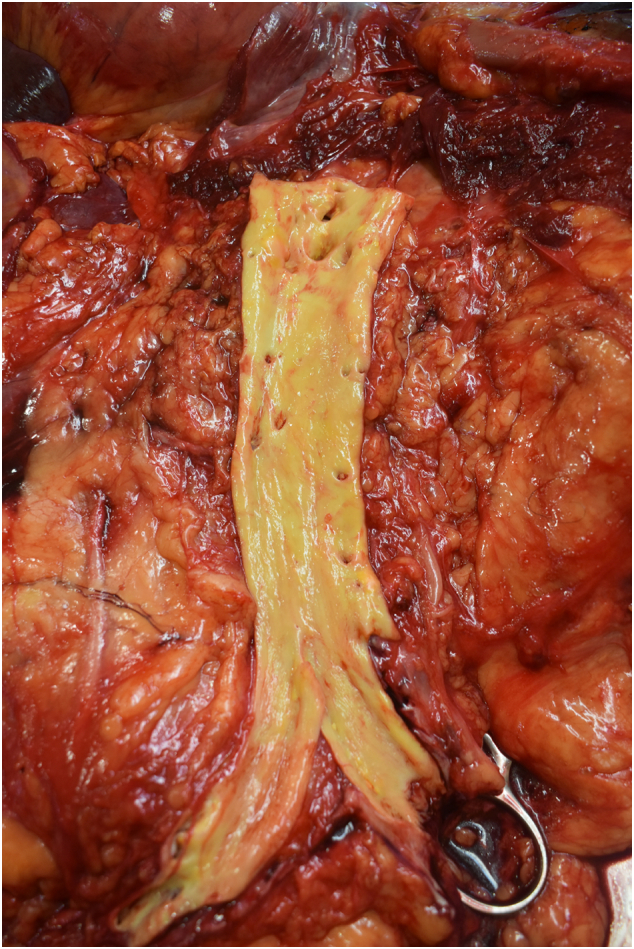
Necropsy study, cutting of the abdominal aorta.

**Fig. 3:**
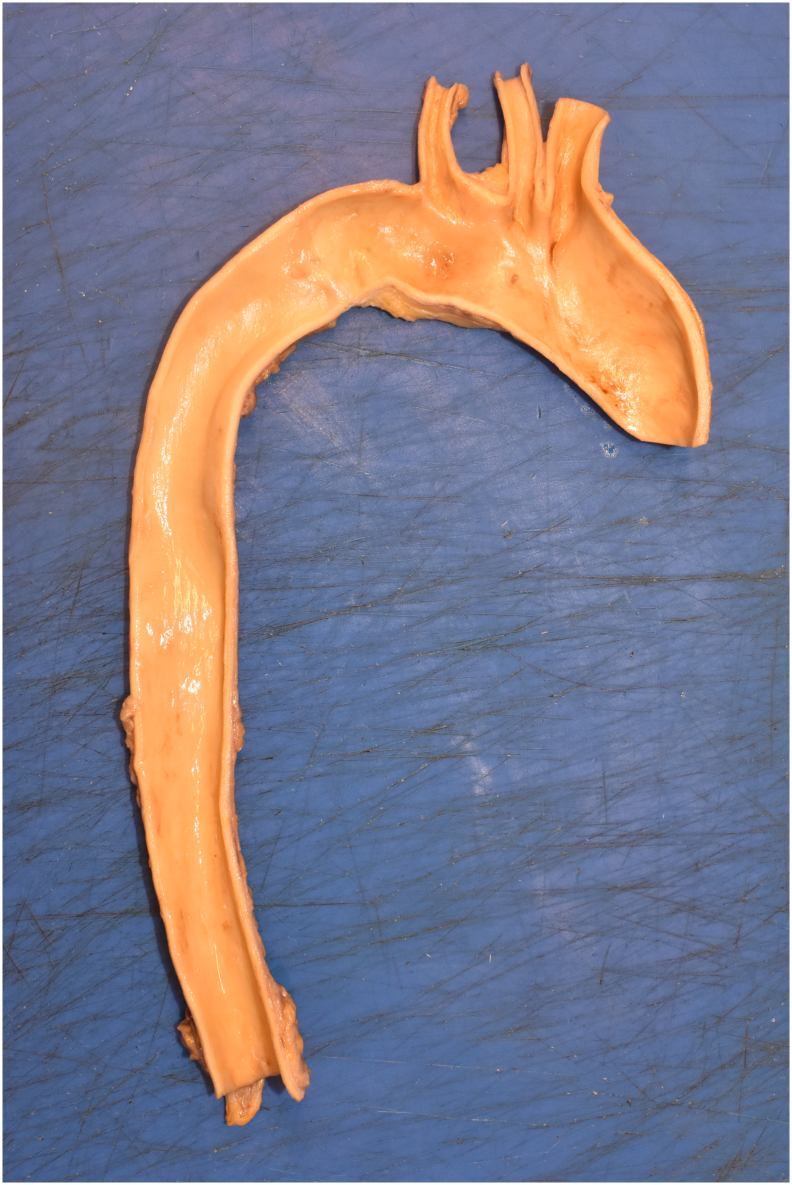
Necropsy study, the ascending aorta.

**Fig. 4:**
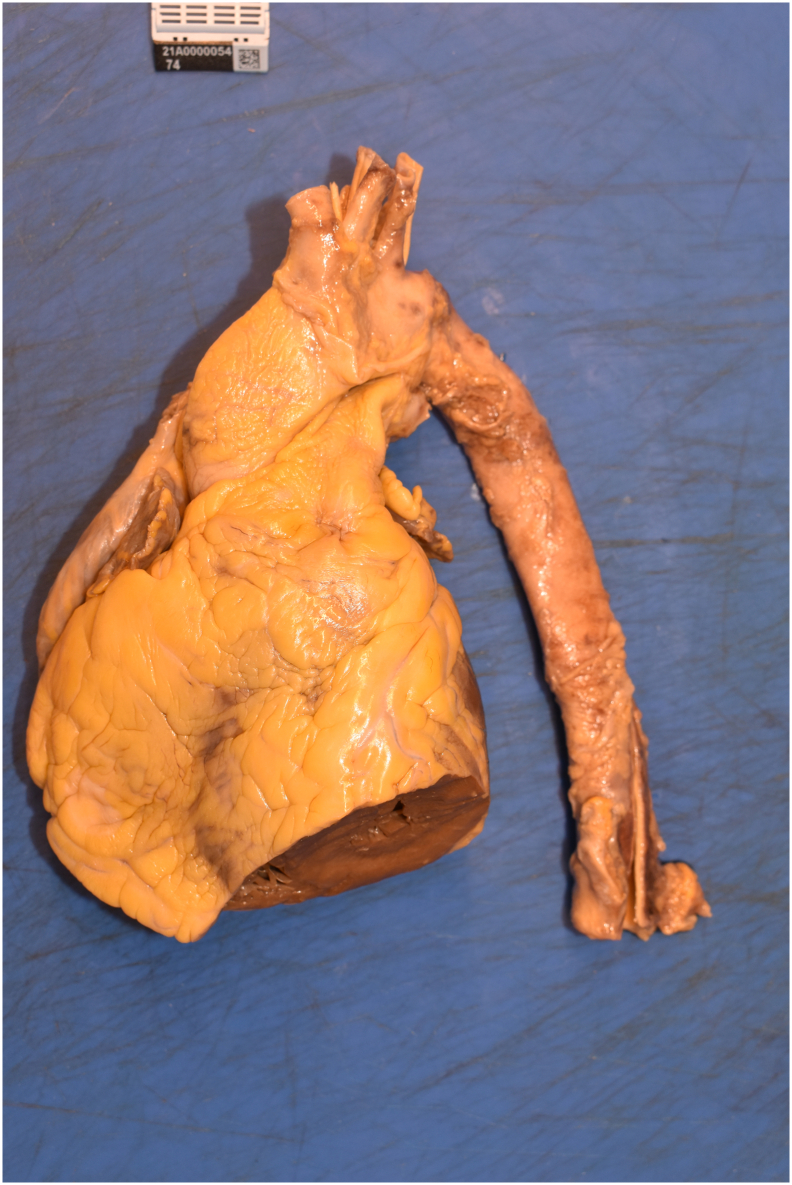
Necropsy study, cutting of the thoracic and ascending aorta.

The coronary angiography was performed by the senior interventional cardiologists. A 6-Fr sheath was introduced into the right femoral artery and another 6-Fr sheath in the right femoral vein. A JL 4 guide catheter 6-Fr was placed over the wire into the ascending aorta, and the left coronary artery was cannulated; it was patent, and there were non-obstructive coronary arteries (Video 1). Then, the catheter was exchanged for a JR 4 guide catheter 6-Fr, while the right coronary artery was cannulated, an image of probable aortic dissection was observed (Fig. 1). The right coronary artery was patent and without angiographic stenosis (Video 2). Given the persistent asystole despite a prolonged advance cardiopulmonary resuscitation and the possibility of aortic dissection, a prompt in-room heart team discussion was performed with the senior cardiac surgeons. It was decided to stop and withdraw potentially life-sustaining treatment due to futility. Of the note, the SARS-CoV-2 RT-PCR test was negative. In addition, genetic testing and necropsy were performed with the consent of his family. The genetic testing reported a negative result for explaining the sudden cardiac death. On the other hand, the aorta in the necropsy study revealed some mild atherosclerotic plaques but without either aneurysm or thrombosis (Fig. 2, Fig. 3, Fig. 4). The coronary arteries were reported as with patency. In the proximal left anterior descending artery (LAD), the intima layer presented a thickness that decreased 50 % of the luminal area, with mild calcification and macrophages but without complicated acute plaques such as rupture, erosion, or thrombus. For the rest, the necropsy study was reported as without relevant pathologies. This case report has been reported in line with SCARE 2020 criteria [2].

## 3 Clinical discussion

The false-positive ascending dissections artifacts have been reported in computed tomography (CT) and transesophageal echocardiogram (TEE) [3], [4]. Although CT imaging is highly sensitive and specific for aortic dissections, there is a potential for false-positive ascending dissections (Stanford type A). Besides, a cohort study reported that 11.3 % of false-positive suspicion of aortic dissections, 10 % was based on CT imaging. [5]. Motion artifacts simulating ascending aortic dissection occur frequently on conventional CT. Their position is predictable and is related to systolic aortic motion from the left anterior to the right posterior position [6]. Nonetheless, in our case, the systolic aortic motion theory cannot explain this because the patient was under cardiac arrest. Hence, the best possible explanation for the image of pseudo-aortic dissection is the abnormal hemodynamic flow in cardiac arrest. A prospective observational single-center study of patients with cardiac arrest, arterial and venous pressures was recorded continuously while receiving advanced cardiac life support, reported that both arterial and venous dominant hemodynamic changes were observed during cardiopulmonary resuscitation. However, venous pressure was dominant to arterial, mainly when mechanical cardiac compressions were used [7]. Moreover, there was an important patients' venous pressure change in the iliac vein, suggesting that the hemodynamic flow might be backward during cardiopulmonary resuscitation. Therefore, in the setting of this abnormal hemodynamic flow, the injection of contrast may have an abnormal distribution and flow in the aorta creating an image of pseudo-aortic dissection. Interestingly, the site of dissection was not clear on the still frame, which could have provided a clue of the false-positive aortic dissection. In addition, the bedside echocardiography did not observe any sign of aortic dissection. Besides, the coronary angiography showed mild non-obstructive coronary disease in the LAD, which in the necropsy study reported moderate stenosis (50 %), suggesting that the abnormal hemodynamic flow produced in cardiac arrest can downgrade the angiographic stenosis. Nevertheless, more research is required to elucidate the mechanism of the false-positive aortic dissection in this scenario.

In contrast, both arterial and venous thromboembolic events and immune thrombocytopenia have been reported with adenovirus vector vaccine against COVID-19 such as ChAdOx1 and Ad26.COV2.S [8], [9]. However, the necropsy study showed no thrombosis at any site. Given the family history of sudden cardiac death, a genetic analysis was performed with 72 gene panels of sudden cardiac death and connective tissue disease, also including 559 genes related to cardiomyopathies, which was negative. Nonetheless, we cannot rule out a rare cardiomyopathy not included in the gene panel. Ultimately, there are some case reports of a variety of acute coronary syndrome after vaccination against COVID-19 with both mRNA and DNA vaccines, from thrombosis [10], [11] to abnormal wall motion with non-obstructive coronary arteries [12]. In this regard, the authors of these case reports mention the Kounis syndrome as a cause of chest pain in patients admitted to the catheterization laboratory with an ongoing allergic reaction [13]. This notwithstanding is probably not in our case due to the lack of allergic reactions in his medical history. Since coronary artery disease is frequent in daily practice and mass vaccination against COVID-19 took place. It seems more likely that, in our case, the COVID-19 vaccine is an innocent bystander and not responsible for the cardiac demise. Furthermore, many studies, such as the Japanese Coronary Spasm Association multicenter registry, have proved that coronary vasospasm is a cause of transient ST-segment changes, episodes of ventricular arrhythmia, and sudden cardiac arrest [14]. Coronary vasospasm can trigger a vulnerable plaque with the development of myocardial ischemia with subsequent sudden cardiac death [15]. In this regard, this is highly probably our case, considering that the necropsy study reported plaque stenosis of 50 % in the proximal left anterior descending artery.

## 4 Conclusion

A variety of other artifacts mimicked aortic dissection have been reported in CT and ETT, which can be identified by their position that is predictable and is related to systolic aortic motion from the left anterior to the right posterior position. So far, to the best of our knowledge, this is the first case report of a pseudo-aortic dissection in coronary angiography. Although the exact mechanism of this false-positive aortic dissection remains unknown, the best possible explanation is that the abnormal hemodynamic flow in cardiac arrest during cardiopulmonary resuscitation may generate an abnormal distribution and flow of the contrast in the aorta creating an image of pseudo-aortic dissection, especially in those with mechanical chest compressions. Of the note, the site of dissection was not clear on the still frame, which could have provided a clue of the false-positive aortic dissection. In addition, the bedside echocardiography did not observe any sign of aortic dissection. Furthermore, this abnormal hemodynamic flow can downgrade the angiographic stenosis, the coronary angiography showed mild non-obstructive coronary disease in the LAD, which in the necropsy study reported moderate stenosis (50 %). Therefore, the operators should be aware of these entities during coronary angiography in cardiac arrest with mechanical chest compressions to avoid diagnostic errors in clinical practice.

The following are the supplementary data related to this article.

Video 1 Left coronary angiography. Video 1

Video 2 Right coronary angiography and image of aortic dissection. Video 2

## Consent

Written informed consent was obtained from the patient for publication of this case report and accompanying images. A copy of the written consent is available for review by the Editor-in-Chief of this journal on request.

## Availability of data and material

Any further data and material can be received through contacting the corresponding author.

## Code availability

Not applicable.

## Provenance and peer review

Not commissioned, externally peer-reviewed.

## Ethical approval

The ethics committee simply required the written statement of consent by the patient.

## Funding

None declared.

## Guarantor

Artemio García-Escobar.

## Research registration number

Not applicable.

## CRediT authorship contribution statement

Artemio García-Escobar: Responsible for the concept design, writing and editing of all the manuscript including the conclusion; Silvio Vera-Vera: writing and editing abstract, and data curation (Figures and Videos); Alfonso Jurado-Román: Writing and editing case presentation; Santiago Jiménez-Valero: Writing and editing case presentation; Guillermo Galeote: Writing and editing discussion; Raúl Moreno: Writing and editing discussion.

## Declaration of competing interest

The authors declare no competing interests.

## References

[1] Batra P., Bigoni B., Manning J., Aberle D.R., Brown K., Hart E., Goldin J. (Mar-Apr 2000). Pitfalls in the diagnosis of thoracic aortic dissection at CT angiography. Radiographics.

[2] Agha R.A., Franchi T., Sohrabi C., Mathew G., for the SCARE Group (2020). The SCARE 2020 guideline: updating consensus Surgical CAse REport (SCARE) guidelines. Int. J. Surg..

[3] Yamane Y., Uchida N., Mochizuki S., Furukawa T., Yamada K. (2016 Jul). Pseudo-dissection of aorta: wall-motion artifact mimicking aortic dissection with a patent false lumen. Eur. Heart J. Cardiovasc. Imaging.

[4] Watke C.M., Clements F., Glower D.D., Smith M.S. (1998 Apr). False-positive diagnosis of aortic dissection associated with femoral cardiopulmonary bypass. Anesthesiology.

[5] Raymond C.E., Aggarwal B., Schoenhagen P., Kralovic D.M., Kormos K., Holloway D., Menon V. (2013 Dec). Prevalence and factors associated with false positive suspicion of acute aortic syndrome: experience in a patient population transferred to a specialized aortic treatment center. Cardiovasc. Diagn. Ther..

[6] Duvernoy O., Coulden R., Ytterberg C. (Jul-Aug 1995). Aortic motion: a potential pitfall in CT imaging of dissection in the ascending aorta. J. Comput. Assist. Tomogr..

[7] J Koyama, Matsuyama T., Inoue Y. (2019 Apr). Blood flow forward into the artery and backward into the vein during chest compression in out-of-hospital cardiac arrest. Resuscitation.

[8] Douxfils J., Favresse J., Dogné J.M., Lecompte T., Susen S., Cordonnier C., Lebreton A., Gosselin R., Sié P., Pernod G., Gruel Y., Nguyen P., Vayne C., Mullier F. (2021 Jul). Hypotheses behind the very rare cases of thrombosis with thrombocytopenia syndrome after SARS-CoV-2 vaccination. Thromb. Res..

[9] Bayas A., Menacher M., Christ M., Behrens L., Rank A., Naumann M. (2021 May 1). Bilateral superior ophthalmic vein thrombosis, ischaemic stroke, and immune thrombocytopenia after ChAdOx1 nCoV-19 vaccination. Lancet.

[10] Tajstra M., Jaroszewicz J., Gąsior M. (2021 May 10). Acute coronary tree thrombosis after vaccination for COVID-19. JACC Cardiovasc. Interv..

[11] Maadarani O., Bitar Z., Elzoueiry M., Nader M., Abdelfatah M., Zaalouk T., Mohsen M., Elhabibi M. (2021 Aug 11). Myocardial infarction post COVID-19 vaccine - coincidence, Kounis syndrome or other explanation - time will tell. JRSM Open.

[12] Özdemir İ.H., Özlek B., Özen M.B., Gündüz R., Bayturan Ö. (2021 Oct). Type 1 Kounis syndrome induced by inactivated SARS-COV-2 vaccine. J. Emerg. Med..

[13] Marchesini D., Esperide A., Tilli P., Santarelli L., Covino M., Carbone L., Franceschi F. (2020 Nov). Allergic acute coronary syndrome: a case report with a concise review. Eur. Rev. Med. Pharmacol. Sci..

[14] Takagi Y., Yasuda S., Tsunoda R., Ogata Y., Seki A., Sumiyoshi T., Matsui M., Goto T., Tanabe Y., Sueda S., Sato T., Ogawa S., Kubo N., Momomura S., Ogawa H., Shimokawa H., Japanese Coronary Spasm Association (2011 Jun). Clinical characteristics and long-term prognosis of vasospastic angina patients who survived out-of-hospital cardiac arrest: multicenter registry study of the Japanese Coronary Spasm Association. Circ. Arrhythm Electrophysiol..

[15] Marzilli M., Huqi A. (2012 Feb 14). Coronary vasospasm and coronary atherosclerosis: do we have to choose?. J. Am. Coll. Cardiol..

